# Newly Designed Specula for Laryngomicroscopy

**DOI:** 10.1155/DTE.3.73

**Published:** 1996

**Authors:** Akihiro Shiotani, Hiroyuki Fukuda, Masahiro Kawaida, Toshiyuki Kusuyama, Hideki Nakagawa, Atsushi Kawasaki, Jin Kanzaki

**Affiliations:** 1 Department of Otolaryngology School of Medicine Keio University 35 Shinanomachi, Shinjuku-ku Tokyo Japan; 2 Tokyo Metropolitan Ohtsuka Hospital Tokyo Japan

## Abstract

We produced specula for laryngomicroscopy to observe blind spots in the operating field. Use of these specula has facilitated detailed observation of the lower surface of the false vocal folds, laryngeal ventricle, and subglottis, which were previously in blind spots. The specula are useful in the following ways: 1) clarifying blind spots for improved diagnosis and providing more accurate surgical margins; 2) observing the lower lips of the vocal folds in phonosurgery; and 3) Vaporizing with laser reflection. The specula are cheap and easy to use and are well worth considering for application to laryngomicroscopy.


*Diagnostic and Therapeutic Endoscopy*, Vol. 3, pp. 73–78
Reprints available directly from the publisher
Photocopying permitted by license only

© 1996 OPA (Overseas Publishers Association)
Amsterdam B.V. Published in The Netherlands
by Harwood Academic Publishers GmbH
Printed in Singapore

### Newly Designed Specula for Laryngomicroscopy

AKIHIRO SHIOTANI*,†, HIROYUKI FUKUDA, MASAHIRO KAWAIDA, TOSHIYUKI KUSUYAMA, HIDEKI NAKAGAWA, ATSUSHI KAWASAKI and JIN KANZAKI

*Departments of Otolaryngology, School of Medicine, Keio University (A.S., H.F., T.K., H.N., A.K., J.K.) and Tokyo Metropolitan Ohtsuka Hospital (M.K.) Tokyo, Japan*

*(Received 28 July 1995; In final form 25 March 1996)*

**We produced specula for laryngomicroscopy to observe blind spots in the operating field. Use of these specula has facilitated detailed observation of the lower surface of the false vocal folds, laryngeal ventricle, and subglottis, which were previously in blind spots. The specula are useful in the following ways: 1) clarifying blind spots for improved diagnosis and providing more accurate surgical margins; 2) observing the lower lips of the vocal folds in phonosurgery; and 3) Vaporizing with laser reflection. The specula are cheap and easy to use and are well worth considering for application to laryngomicroscopy.**

*Keywords:* Blind spots, laryngomicroscopy, laser surgery, mirror, phonosurgery

### INTRODUCTION

The development and spread of laryngomicroscopy has facilitated the diagnosis and treatment of microscopic lesions of the larynx. Laryngomicroscopy is effective for detailed observation of the upper surface of the vocal and false vocal folds. This can be done by simply inserting a laryngoscope and widening the area of view by tilting the laryngoscope to the left or right or by pressing down the folds with forceps. However, because laryngomicroscopy involves an extremely deep and narrow area of view, blind spots still exist even when these maneuvers are added. The subglottis, laryngeal ventricle, and lower surface of the false vocal folds are difficult to observe in most cases.

While laryngomicroscopy is performed usually by observation from the upper surface of the vocal folds only, subglottic observation would seem useful, considering the fact that the vocal mucosal wave starts near the lower lips of the vocal folds.

For these reasons, we produced laryngomicroscopic specula to observe potential blind spots during laryngomicroscopy and have found them to be clinically useful.

### PRODUCTION OF THE SPECULA

The laryngomicroscopic specula, which were produced in cooperation with Nagashima Medical Instruments Co., Ltd., are of two different sizes (Fig. 1,

*Corresponding author.
†Present address: Department of Otolaryngology, School of Medicine, Keio University, 35 Shinanomachi, Shinjuku-ku, Tokyo 160, Japan.

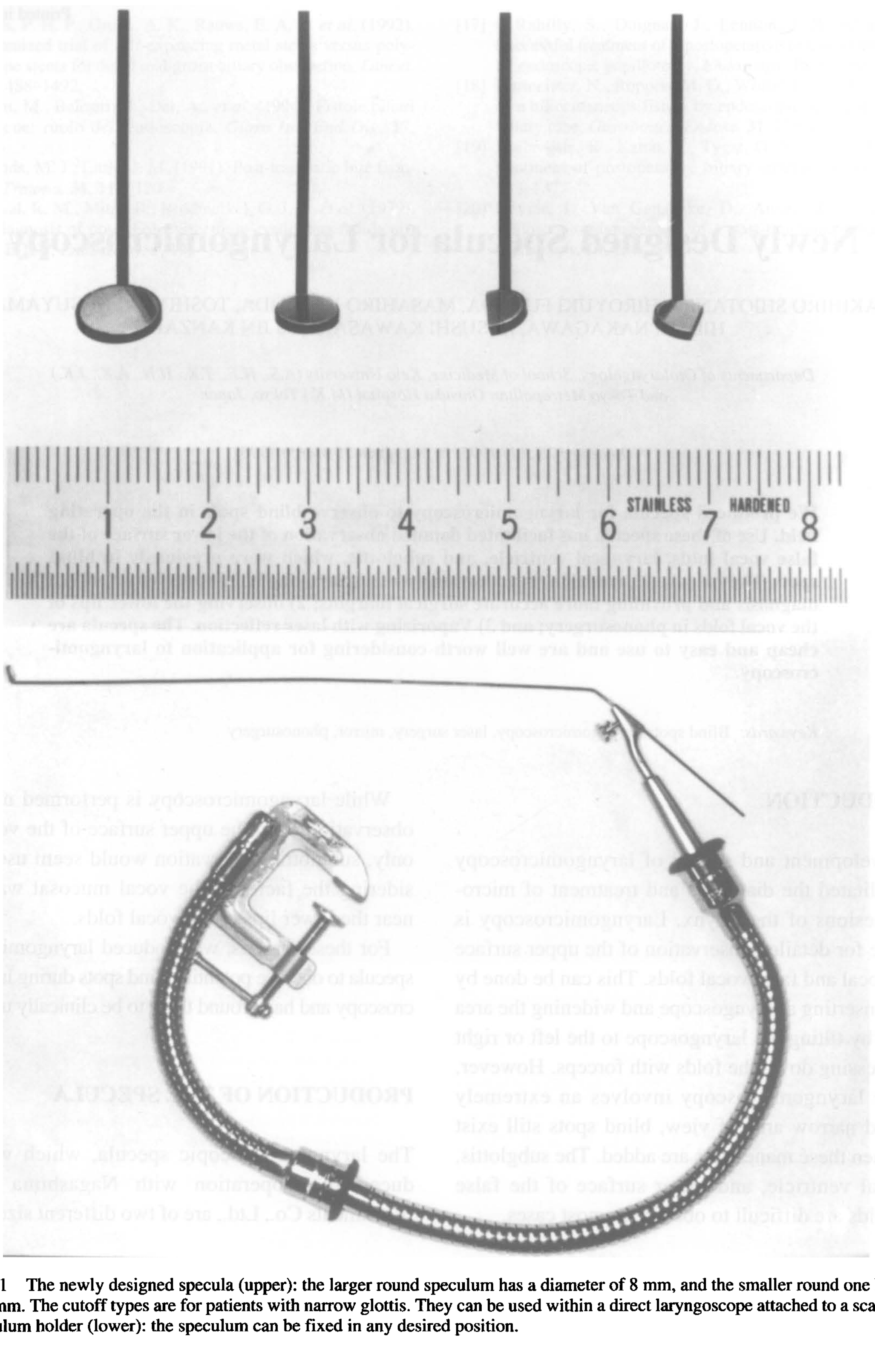

FIGURE 1 The newly designed specula (upper): the larger round speculum has a diameter of 8 mm, and the smaller round one has a diameter of 6 mm. The cutoff types are for patients with narrow glottis. They can be used within a direct laryngoscope attached to a scalpel holder. The speculum holder (lower): the speculum can be fixed in any desired position.

upper). The larger one is 8 mm in diameter, and the smaller one is 6 mm in diameter. Both can be used inside a direct laryngoscope. The mirrored surface is chromium-plated with an aluminum material and has a slender black brass-plated handle 0.7 mm in diameter and 32 cm in length. The angle between the long axis of the handle and the mirror is 50°. In addition, for patients with narrow glottis, we produced a speculum with one-third of the mirror on the left side cut off, and another with the right side cut off. In practice, use with a direct laryngoscope is made easier by attaching the speculum to a scalpel holder for laryngomicroscopy and bending the handle to an appropriate angle.

Further, we designed a speculum holder so that the speculum can be fixed without using the hands (Fig. 1, lower). The holder is screwed to an operating table, and the handle of the speculum is attached to the holder, which can be bent freely to the desired position. With this device, other procedures can be performed while looking at the speculum fixed inside the direct laryngoscope.

### CLINICAL APPLICATIONS

### Observation of Blind Spots: Case 1: Right Glottic Tumor

The tumor appeared to be localized to the glottis from the normal field of view. Observation of the laryngeal ventricle with the speculum revealed tumorous changes of the mucous membrane deep in the laryngeal ventricle confirming tumor infiltration (Fig. 2). The diagnosis in this patient was T2 right glottic squamous cell carcinoma.

### Application in Phonosurgery: Case 2: Left Local Fold Polyp

The speculum was fixed at the lower side of the vocal fold using the holder, and the polyp was resected (Fig. 3). Use of the speculum provided detailed observation of the area near the lower lip and facilitated surgery with combined observations from above and below the vocal fold, providing more information.

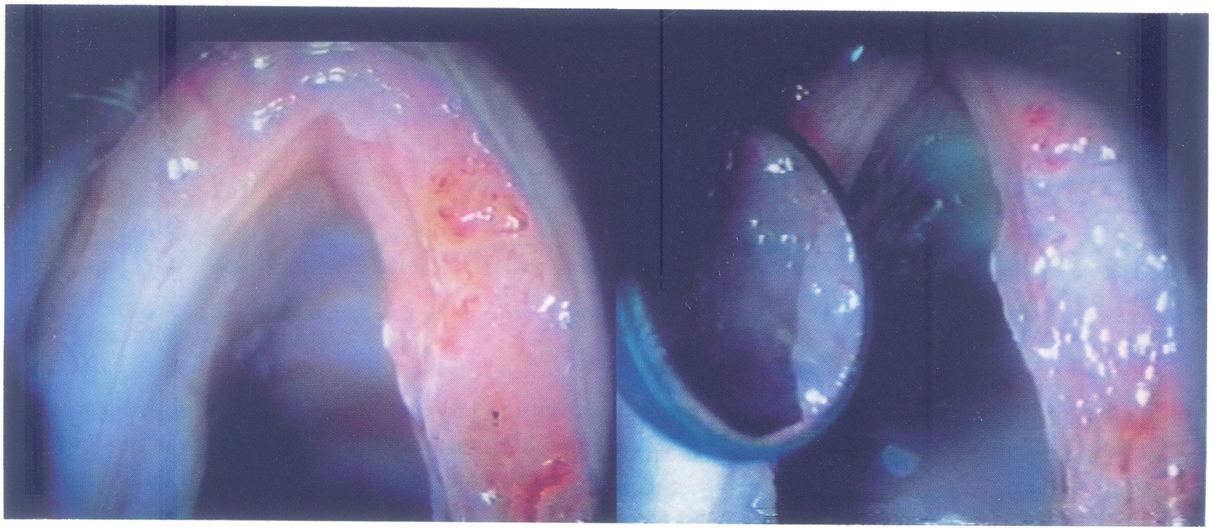

FIGURE 2 Case 1: a right glottic tumor (left). Observation of the laryngeal ventricle with the speculum reveals tumor infiltration into the ventricle (right).

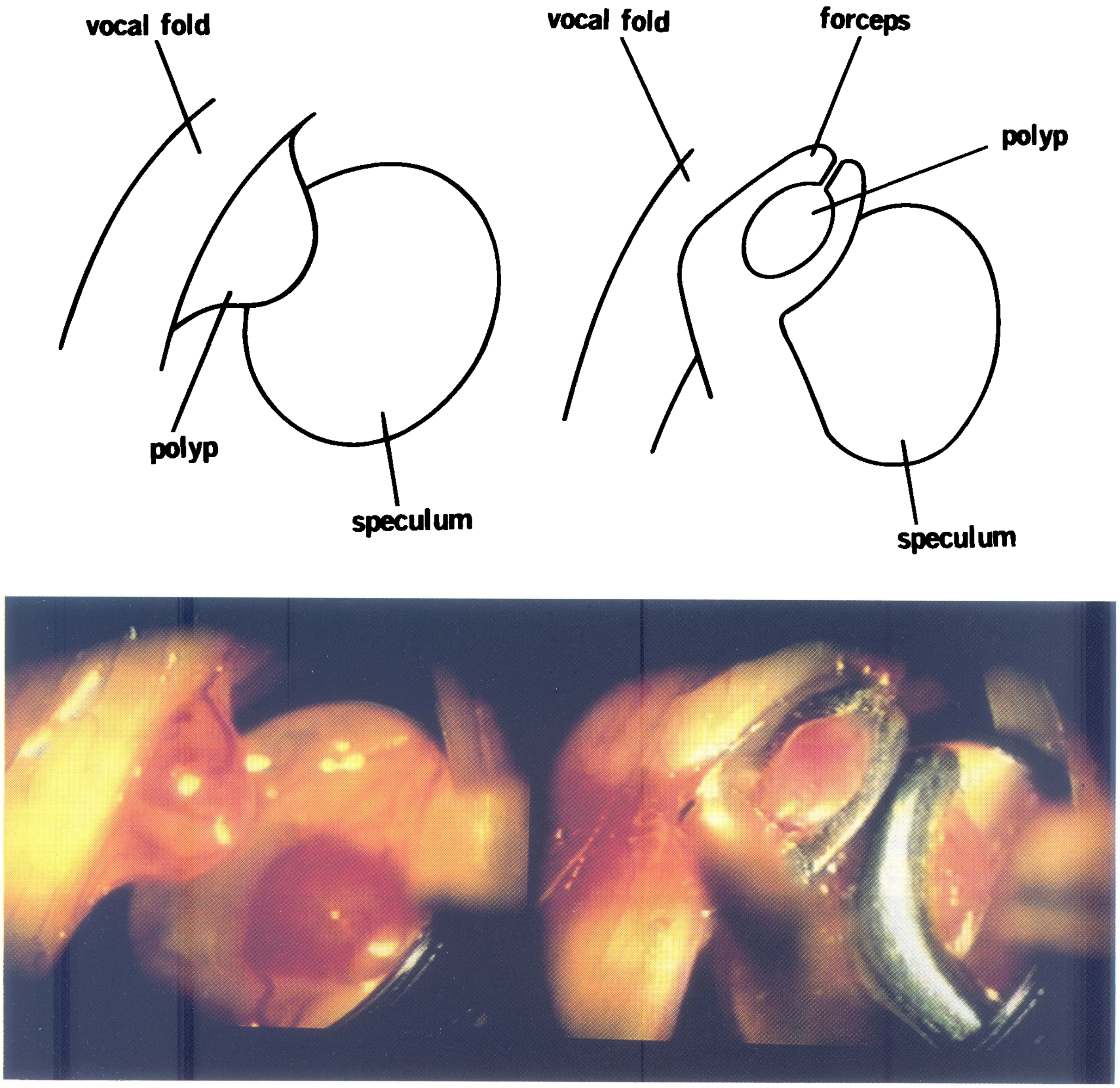

FIGURE 3 Case 2: A left vocal fold polyp (left). The speculum provides more information by allowing observations from above and below the vocal fold to be combined (right).

**Application to Laser Surgery: Case 3: Recurrent Laryngeal Papilloma**

A $CO_2$ laser was used for surgery. Although the region as observed from above had been vaporized completely, the speculum detected a skipped area from the laryngeal ventricle to the lower surface of the false vocal fold. As this region was shadowed by the false vocal fold and appeared difficult to irradiate directly with the laser, the speculum was used to reflect the laser beam to vaporize the tumor completely (Fig. 4).

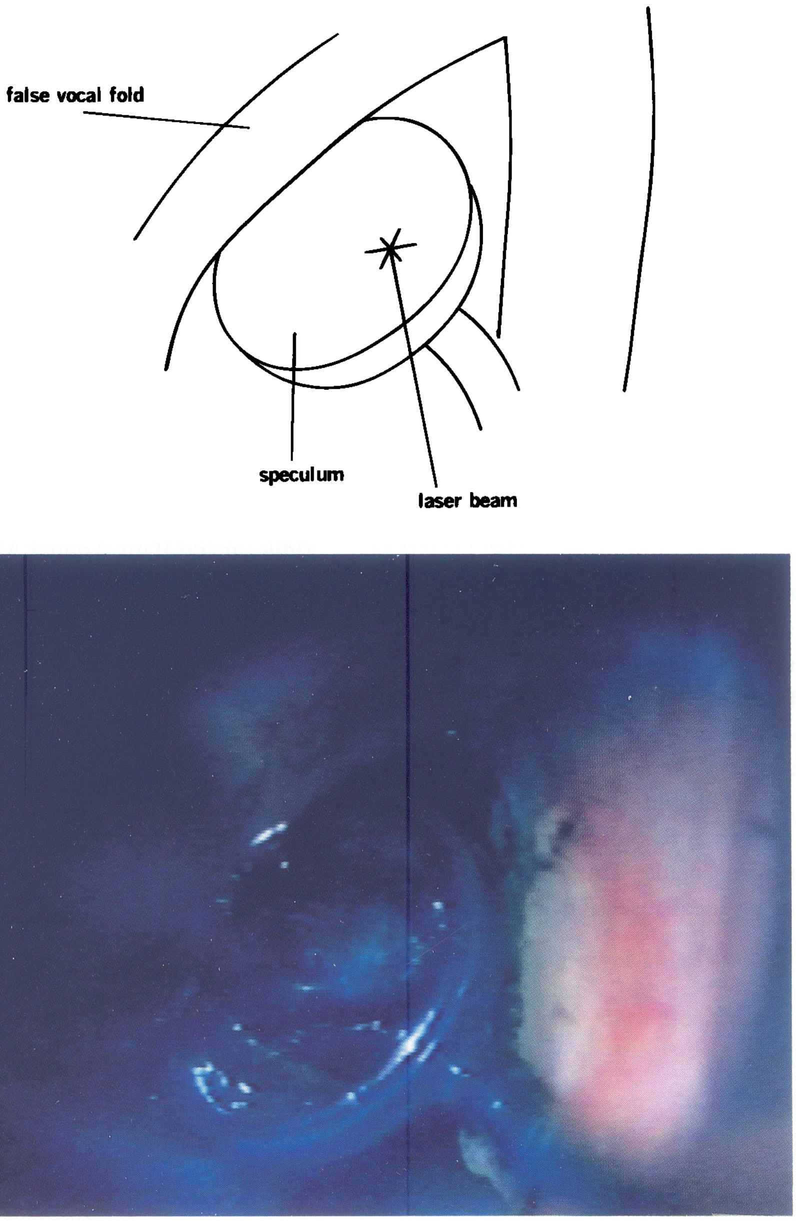

FIGURE 4 Case 3: A case of a recurrent laryngeal papilloma. A $CO_2$ laser was directed to the region shadowed by the false vocal fold by reflecting it with the speculum.

### DISCUSSION

There are devices for observating blind spots during laryngomicroscopy with Jaco [1] producing a small mirror for examination of the subglottis and Kantor *et al.* [2] reporting the use of a mirror of 10 mm in diameter to observe the laryngeal ventricle and subglottis, together with a video microlaryngoscope. However, these devices have never been applied to phonosurgery or laser surgery. Furthermore, the latter mirror is specific for video microlaryngoscopy and seemed inappropriate for use inside a conventional direct laryngoscope due to its size. While laser reflectors that can be used inside an ordinary direct laryngoscope have been marketed by Storz, it is not certain whether they are useful for the observation of blind spots or phonosurgery.

Hence, we have produced our own specula on the basis of the concepts stated previously and found them useful in the following clinical applications.

1. Blind spots including the lower surface of the vocal folds, laryngeal ventricle, and subglottis can be observed easily. The specula are useful for the accurate assessment of the extent of tumor infiltration. They also provide an accurate estimation of the early stages of ventricular and subglottic cancers. In addition, the surgical margin can be confirmed to assure complete resection.

2. In phonosurgery, pathologic regions can be observed in detail from the subglottis. This permits detailed observation of the region near the lower lips, that is the area where the mucosal wave starts, ensuring a satisfactory surgical result. Thus, a combination of usual observation from above and specular observation from the subglottis provides more information and facilitates the performance of surgery that allows full appreciation of mucosal wave function to be made. This contributes to the performance of an ideal resection with a minimum of intervention.

3. Laser irradiation of inaccessible areas under direct view is possible using reflection.

As another method for observing blind spots in laryngomicroscopy, a rigid laryngotelescope can be used inside a direct laryngoscope. A rigid laryngotelescope ought to be able to be used in the same way as a speculum to observe details of the lower surface of the false vocal folds, laryngeal ventricle, and subglottis; however, specular observation has advantages in terms of price and simplicity of use, in that treatment can be performed simultaneously.

### *Acknowledgments*

This paper was presented at the 6th Annual Meeting of the Laryngological Society of Japan, Saga, March 11, 1994; at the 95th Annual Meeting of the Otolaryngological Society of Japan, Niigita, May 19, 1994; at the 3rd International Symposium on Phonosurgery, Kyoto, June 27, 1994; and at the video symposium of 11th Congress of Pan-Pacific Surgical Association Japan Chapter, Okinawa, November 24, 1994.

### *References*

[1] Jako, G. L. (1970). Laryngoscopy for microscopic observation, surgery, and photography, *Arch Otolaryngol*, **91**, 196–199.

[2] Kantor, E. A., Berci, G., Partlow, E., *et al.* (1991). Ancillary instruments for the video microlaryngoscope, *Ann Otol Rhinol Laryngol*, **100**, 317–919.

